# Unraveling Venetoclax Resistance: Navigating the Future of HMA/Venetoclax-Refractory AML in the Molecular Era

**DOI:** 10.3390/cancers17091586

**Published:** 2025-05-07

**Authors:** Theodora Chatzilygeroudi, Theodoros Karantanos, Vasiliki Pappa

**Affiliations:** 1 Second Department of Internal Medicine and Research Unit, Hematology Unit, National and Kapodistrian University of Athens School of Medicine, Attikon University Hospital, 12462 Athens, Greece; thchatzilygeroudi@gmail.com; 2 Division of Hematologic Malignancies and Bone Marrow Transplantation, Department of Medical Oncology, The Johns Hopkins University School of Medicine, Baltimore, MD 21205, USA; tkarant1@jhmi.edu

**Keywords:** venetoclax, resistance, AML, clinical trials

## Abstract

Acute myeloid leukemia (AML) has traditionally been linked to a poor prognosis, particularly in older patients who are ineligible for intensive chemotherapy. The advent of Venetoclax, a powerful oral BH3 mimetic targeting anti-apoptotic protein BCL2, has significantly advanced AML treatment. Its combination with the hypomethylating agent azacitidine (AZA/VEN) has become a standard treatment for this group of AML patients, demonstrating a 65% overall response rate and a median overall survival of 14.7 months, compared to 22% and 8 months with azacitidine monotherapy, respectively. However, resistance and relapses remain common, representing a significant clinical challenge. Recent studies have identified molecular alterations, such as mutations in *FLT3-ITD*, *NRAS/KRAS*, *TP53*, and *BAX*, as major drivers of resistance. Additionally, other factors, including metabolic changes, anti-apoptotic protein expression, and monocytic or erythroid/megakaryocytic differentiation status, contribute to treatment failure. Clinical trials are exploring strategies to overcome venetoclax resistance, including doublet or triplet therapies targeting IDH and FLT3 mutations; novel epigenetic approaches; menin, XPO1, and MDM2 inhibitors; along with immunotherapies like monoclonal antibodies and antibody–drug conjugates. A deeper understanding of the molecular mechanisms of resistance through single-cell analysis will be crucial for developing future therapeutic strategies.

## Simple Summary

The combination of Venetoclax, a BLC2 inhibitor, with hypomethylating agents (HMA/VEN) has become a standard treatment for older AML patients unfit for intensive chemotherapy. However, resistance and relapses are common, and significant challenges remain. The main genetic alterations associated with resistance are *TP53*, *FLT3-ITD*, and *RAS* mutations. Non-genetic resistance mechanisms have also been described, including alterations in apoptotic proteins, metabolic changes, and differentiation status. Ongoing trials are exploring combination therapies and novel approaches to overcome resistance. Advanced technology, such as single-cell analysis, will be crucial for understanding resistance mechanisms and improving outcomes for HMA/VEN-refractory AML patients.

## 1. Introduction

Acute myeloid leukemia (AML) is a heterogeneous hematologic malignancy associated with a generally poor prognosis, and approximately 70% of patients are diagnosed over the age of 65. While advancements in targeted therapies have significantly improved outcomes, resulting in more effective and personalized treatment options, a substantial unmet clinical need persists.

The introduction of venetoclax (VEN), a selective antagonist of the B-cell lymphoma 2 (BCL2), has markedly transformed the treatment landscape for AML. While VEN monotherapy demonstrated an overall response rate of only 19% [1], its combination with hypomethylating agents (HMAs) has provided a more effective and less intensive therapeutic option, particularly for elderly or unfit patients. With response rates around 65% and with a 2-year overall survival (OS) of 37.5% in the pivotal VIALE-A study [2,3], along with promising results in real-world data [4,5], this combination has effectively addressed the therapeutic gap for patients unfit for chemotherapy at diagnosis but not elderly enough to only be given a 15–30% chance of response and 10 months of survival with HMA monotherapy [4,6,7]. However, following their initial response to treatment, patients typically relapse after a median of 18 months, indicating that this combination is not a curative therapy for the majority of individuals with AML [2].

In contrast to the promising outcomes of HMA/VEN treatment, relapsed or refractory AML (R/R-AML) following HMA/VEN treatment is associated with an extremely poor prognosis [8]. Thus, addressing primary and secondary resistance to venetoclax-based regimens is crucial as we enter the era of molecular targeted therapies for AML. Recent studies have identified both genetic and non-genetic factors contributing to the development of resistance, deepening our understanding and paving the way for emerging novel therapeutic strategies. This review will focus on the primary mechanisms of resistance and explore potential therapeutic approaches to overcome them.

## 2. Venetoclax Mechanism of Action

### 2.1. Intrinsic Apoptosis Mechanism: BCL2 Family Proteins in AML

AML cells originate from myeloid precursor cells and are characterized by impaired differentiation and resistance to apoptosis. Genetic mutations drive the clonal expansion of malignant cells by providing a survival advantage [9,10]. As expected, the expansion of dominant subpopulations is often linked to alterations in apoptotic signaling; thus, targeting apoptosis could offer a therapeutic approach in AML that operates independently of specific mutations or patient characteristics [10].

**Figure 1:**
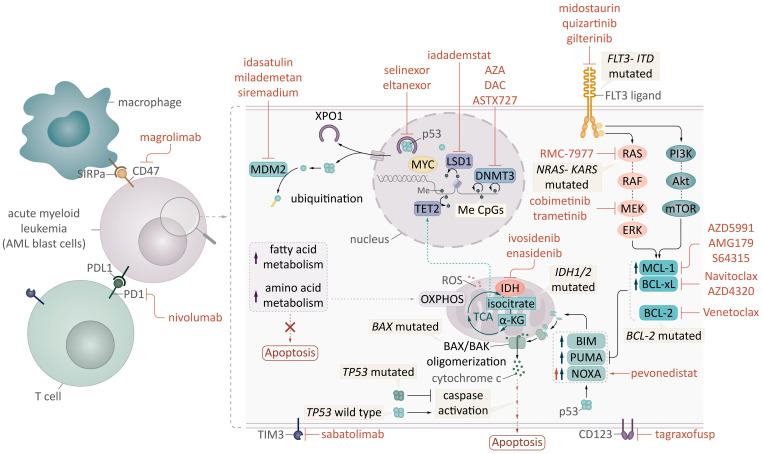
Mechanisms of venetoclax resistance and current therapeutic strategies with new targeted combinations in acute myeloid leukemia (AML). FLT3: FMS-like tyrosine kinase 3; ITD: internal tandem duplication; MYC and p53: transcription factors; LSD1: lysine-specific histone demethylase; Me: methylation; CpG: CG sites, DNMT3A: DNA methyltransferase 3A; TET2: tet methylcytosine dioxygenase 2; a-KG: alpha-ketoglutarate; 2-HG: 2-hydroxyglutarate; ROS: reactive oxygen species; OXPHOS: oxidative phosphorylation; TCA: tricarboxylic acid cycle; AZA: 5-azacitidine; DAC: decitabine; IDH: isocitrate dehydrogenase, XPO1: exportin 1; MDM2: E3 ubiquitin–protein ligase.

BCL2 is a key regulator of the intrinsic mitochondrial apoptosis pathway and is a member of the homonymous family of proteins, consisting of three categories of proteins that share BCL2 homology motifs (BH1-4): the proapoptotic BH3-only proteins, anti-apoptotic BCL2 proteins, and the proapoptotic BCL2 effector proteins BAX and BAK (Figure 1). The activation of these later effector proteins is responsible for mitochondrial outer membrane permeabilization (MOMP), the most critical event to initiate cytochrome *c*-mediated intrinsic apoptosis. BH3-only proteins function as either sensitizers (e.g., BAD, BIK, HRK, and NOXA) or activators (e.g., BIM, BID, and PUMA) of apoptosis [11,12,13]. Sensitizer proteins do not directly activate the downstream effector proteins (BAX and BAK) but instead prepare the cells for apoptosis by binding to anti-apoptotic BCL-2 proteins, causing the release of BAX or BAK or triggering the activation of BH3-only activator proteins [14]. The activation of the effector protein induces cytochrome *c* release into the cytosol, facilitating the formation of the apoptosome and the activation of caspase-9. Anti-apoptotic proteins, such as BCL2, BCL2A2, MCL1, and BCL2L1 (BCL-xL), sequester pro-apoptotic proteins by binding to the BH3 motif, preventing them from activating BAX and BAK.

Compared to normal myeloid cells, AML cells have an increased threshold of apoptosis due to upregulated anti-apoptotic proteins and repressed BH3-only proteins BIM and NOXA and high levels of BCL2 and MCL1 [10]. Elevated levels of anti-apoptotic proteins of the BCL2 family, including BCL2, play a crucial role in mediating AML survival and are linked to chemotherapy resistance [15,16,17]. BCL2 is known to promote the survival of leukemic stem cells (LSCs) in AML [18], and its inhibition leads to the eradication of quiescent LSCs, highlighting its potential as a critical therapeutic target.

### 2.2. Venetoclax-Induced Death in AML

Due to their central role in maintaining mitochondrial integrity and AML survival, drugs were developed to target BCL2 family proteins. Early BH3 mimetics, such as ABT-737 [19] or ABT-263/Navitoclax [20], targeted multiple BCL-2 family proteins but had disadvantages such as platelet toxicity [21]. The development of ABT-199/Venetoclax revealed that, despite the functional and structural similarities among anti-apoptotic BCL2 proteins, it is possible to selectively target individual BCL2 proteins [22].

VEN is a BH3 mimetic that displaces pro-apoptotic BH3-only proteins from BCL2, facilitating the activation of proapoptotic BAX or BAK proteins to induce apoptosis. Recent studies showed that targeting BCL2 with VEN induces mostly BAX-dependent apoptosis, and the response to VEN is not related to BCL2 expression alone [1,10]. However, indexes accounting for other BCL2 family member expression patterns, such as MCL1 and BCL-xL, can predict the clinical response and remission duration [1,23], indicating that the overall apoptosis sensitivity of LSCs is important for VEN action.

Apart from its direct action in apoptosis, VEN also enhances T cell-mediated cytotoxicity against AML in vivo. Specifically, VEN acts to impair respiratory chain super complex formation. Thus, it promotes the generation of reactive oxygen species (ROS) and boosts the effector function of CD3^+^CD4^−^CD8^−^ double-negative and CD8^+^ T cells [24]. Recent research work characterizing the phenotype and immune checkpoint receptor expression on CD4^+^ and CD8^+^ T cells from AML patients after the first and second cycles of HMA/VEN found an increase in naïve CD8^+^ T cells and TIM-3^+^, CD4^+^, and CD8^+^ T cells, along with a reduction in cytokine-secreting non-suppressive T regulatory cells (Tregs) [25]. Moreover, VEN specifically modulated the immune repertoire by enhancing TIM-3 expression on CD8^+^ T cells and reducing the co-expression of PD-1 and TIM-3 on both CD4^+^ and CD8^+^ T cells [25].

### 2.3. Combination of Venetoclax with HMAs: The Perfect Match

Single-agent HMA regimens are well tolerated and have low treatment-related mortality rates, but they are associated with low response rates (10–50%) and a median survival of just 6–10 months [6,7]. The overexpression of anti-apoptotic proteins (BCL2, BCL-xL, and MCL1), which is linked to chemotherapy resistance in AML, prompted the addition of VEN to low-dose cytarabine (LDAC) or HMA therapy.

Indeed, the ex vivo treatment of clinical AML samples demonstrated synergy between azacitidine (AZA) and VEN at low nanomolar concentrations [13,26,27], and the combination of AZA with BCL2 inhibition was shown to synergistically induce the apoptosis of AML cells [28]. Research into the mechanisms of synergy revealed that AZA treatment in AML cell lines induces pro-apoptotic changes, including a reduction in MCL1 protein level [27,28]. Moreover, through its action on the integrated stress response, AZA primes cells to VEN-induced apoptosis by increasing NOXA levels [29,30]. The combination of AZA with early BH-3 mimetic ABT-737 not only enhanced AML cell killing synergistically but also reduced leukemic cell tissue invasion in vivo, potentially mitigating resistance through niche microenvironment evasion mechanisms in AML [28].

Moreover, the analysis of LSCs from patients treated with AZA/VEN showed the disruption of the tricarboxylic acid cycle, with decreased α-ketoglutarate and increased succinate levels, and in vitro modeling confirmed the reduced glutathionylation of succinate dehydrogenase, thereby suppressing OXPHOS and selectively targeting LSCs. These data suggest that AZA-mediated differentiation of LSCs coupled with VEN-mediated programmed cell death further synergizes each agent’s activity [31]. Additionally, VEN enhances the effectiveness of HMAs by inhibiting the Nrf2 antioxidant pathway, which is activated via HMA treatment. This inhibition leads to increased mitochondrial ROS production and oxidative killing of AML cells [32,33]. Studies have shown that venetoclax disrupts the Nrf2/Keap-1 complex, promoting Nrf2 degradation and reducing antioxidant protein levels, thereby sensitizing cells to oxidative stress [33]. Additionally, combinations of HMA/VEN have been shown to induce ROS accumulation, downregulate pro-survival proteins like Nrf2 and MCL1, and trigger apoptosis in AML cells [34].

Preclinical findings led to the initiation of a phase Ib study [35], followed by the DiNardo et al. phase III trial [2] of combining AZA or DAC with venetoclax in treatment-naïve AML patients aged 65 and older, who were ineligible for standard induction chemotherapy. Moreover, recently reports indicate even higher response rates in AML, MDS, or chronic myelomonocytic leukemia (CMML) treated with a once-weekly low-dose DAC combined with VEN, proposing a non-cytotoxic frequent and sustained HMA exposure in combination with VEN [36].

## 3. Understanding the Mechanisms of Resistance to Venetoclax

### 3.1. BLC2 Family Protein Changes: Facilitating Resistance to Apoptosis

As suggested by prognostic models that account for the expression patterns of other BCL2 family members [1,23], the overall apoptosis sensitivity of LSCs plays a crucial role in determining VEN sensitivity. Therefore, one of the key cellular mechanisms underlying VEN resistance is the alteration in the expression of BCL2 family proteins.

Dependency on other anti-apoptotic proteins, such as BCL2A1, MCL1, and BCL-xL, is linked to primary or secondary resistance [1,13,37,38,39]. Mechanistic studies show that this is primarily due to the sequestration of BH3-only proteins (i.e., BIM) from these anti-apoptotic proteins, preventing them from interacting with BAK/BAX to trigger apoptosis [40]. Through comparative analyses of isogenic AML cell lines sensitive or resistant to VEN, increased stability and higher levels of MCL1 and/or BCL-xL are identified as a major acquired mechanism of VEN resistance [37,41]. Indeed, inhibiting these anti-apoptotic proteins restores VEN sensitivity in resistant AML cell lines [37]. Data from PDX models and human clinical samples confirmed that reduced mitochondrial apoptotic priming due to alterations in BCL2 family proteins characterizes BH3 mimetic resistance [42].

It has been well established that CLL resistance to VEN can arise through mutations in *BCL2* itself, such as the Gly101Val mutation, which impairs or reduces VEN binding [43,44,45,46,47]. In AML, however, resistance-associated point mutations which reduce the affinity of BCL2 for VEN were just very recently reported [48,49]. Specifically, Brown et al. recognized the emergence of on-target *BCL2* variants during VEN treatment, such as *BCL2* Asp103Glu, Phe104Leu, and Val148Leu. These variants were found to be mediators of VEN resistance and rapid emergence of polyclonal variants in AML blasts resistant to VEN dose escalation [49]. Additionally, in patients with CLL, Blombery et al. detected simultaneous BCL2 mutations within CLL cells and BAX mutations in the myeloid compartment of the same patients, indicating lineage-specific adaptation to VEN therapy. The C-terminal BAX mutations abrogated outer mitochondrial membrane localization of BAX and engendered resistance in VEN-induced apoptosis [50]. In line with this, recently, it was shown that 17% of AML patients relapsing after VEN-based therapy have acquired inactivating missense or frameshift/nonsense mutations in the apoptosis effector gene *BAX* [51]. It is of interest that while the *BCL2* Gly101Val mutation primarily impacts drugs targeting BCL2, *BAX* mutations are likely to confer resistance to a broader range of agents that activate the intrinsic apoptotic pathway. In a preclinical study using a genome-wide CRISPR/Cas9 screen in AML cell lines, *BAX*, along with *TP53*, was identified as a key gene whose inactivation leads to VEN resistance [52].

### 3.2. Insights from Clinical Data: Mutation Patterns in HMA/VEN Response and Resistance

Ongoing research has aimed to develop new prognostic scoring systems for patients treated with HMA/VEN combinations, such as the European LeukemiaNet (ELN) genetic risk classifications, as those traditionally based on chemotherapy responses do not effectively predict outcomes for older patients receiving this treatment [53,54]. A pooled analysis of the phase 3 VIALE-A trial and the phase 1b study assessed prognostic stratification using the 2017 and 2022 ELN risk classifications in patients treated with AZA/VEN. Although the outcomes with AZA/VEN were better across all ELN risk groups compared to placebo-AZA, the ELN risk classifications failed to effectively differentiate outcomes in AZA/VEN-treated patients [53]. New molecular signatures based on mutations in *TP53*, *FLT3-ITD*, *NRAS*, and *KRAS* were identified, classifying patients into higher-, intermediate-, and lower-benefit groups. The higher-benefit group comprised slightly over half of the enrolled patients, included those without *TP53* mutations, signaling mutations (*KRAS/NRAS*), or *FLT3-ITD*, corroborating prior clinical and preclinical findings that these mutations are associated with VEN resistance in AML [55,56,57,58,59,60]. The intermediate-benefit group included patients with unmutated *TP53* but with the presence of *FLT3-ITD* and/or *KRAS/NRAS* mutations, showing a median OS of 12.1 months, compared to 26.1 months in the higher-benefit group. Patients in the lower-benefit group included those with *TP53* mutations, in whom AZA/VEN improved response rates compared to placebo-AZA but did not result in prolonged survival, which was only 5.5 months [53]. Similarly, in a pooled analysis of phase II and phase III studies, patients without *TP53* mutations had a response rate of 70% and a median OS of 23.4 months, compared to 41% and 5.2 months in patients with *TP53* mutations [61]. A real-life study from MD Anderson and a DAC/VEN study confirmed these findings, showing a 6–9-month OS for *TP53*-mutated patients, raising concerns about the efficacy of VEN in this group [58,62].

Moreover, among patients in the highest-benefit group, the median OS was notably longer than those with *NPM1* (39.0 months), *IDH2* (36.9 months), *RUNX1* (32.5 months), and *IDH1* (27.7 months) mutations [53]. A subset analysis of the CAVEAT trial confirms that treatment-naive *NPM1*- and *IDH2*-mutant blasts are highly sensitive to VEN monotherapy [63]. After a 7-day VEN pre-phase, 30% of patients achieved more than a 50% reduction in bone marrow blasts. This reduction was particularly pronounced in patients with *NPM1* (56%) and *IDH2* (56%) mutations, while less significant reductions were observed in *TP53* (17%) and *FLT3-ITD* (7%) mutations [63]. In another study, a total of 81 patients were treated with a VEN-based combination, and a complete response was achieved in 64% of patients, with the highest response rates (>80%) observed in those with *NPM1*, *IDH1*, *IDH2*, or *DNMT3A* mutations [56]. Among 18 patients with durable remission lasting over 12 months, *NPM1* and *IDH2* mutations were most frequently observed. Lastly, while *NPM1* mutations were undetectable when assessed by highly sensitive quantitative reverse transcription polymerase chain reaction (RT-qPCR) during remission for up to 24 months, and *IDH2* mutations showed variable molecular persistence [56].

These findings highlight *NPM1* mutations as a strong predictor of a favorable response and durable remission not only to chemotherapy but to VEN-based treatment as well, with *IDH2* mutations also emerging as an important secondary predictor. Conversely, *TP53* mutations are associated with resistance, followed by *FLT3-ITD* and *RAS* mutations as additional predictors of a poor response.

### 3.3. Biological Implications of Mutations Linked to Venetoclax Resistance

#### 3.3.1. TP53 Mutation: The Secrets of the Gatekeeper and Its Partners in Crime

Recently, using genome-wide CRISPR/Cas9 screening led to the identification of p53, BAX, and PMAIP1 (NOXA) as key factors of VEN resistance [52]. The loss of *BAX* or *TP53* inactivation led to resistance by impairing apoptosis execution and increasing reliance on alternative BCL2 family members like BCL2L1. This resistance was also linked to changes in mitochondrial homeostasis and cellular metabolism.

In *TP53* knockout (KO) cells, the levels of TP53 target genes like *PMAIP1*, *PUMA*, and *BAK1* were reduced, while the anti-apoptotic proteins BCL2 and MCL1 were lower across all *TP53* knockout lines, correlating with increased BCL2L1 (BCL-xL) expression, which may contribute to VEN resistance [52]. Moreover, loss-of-function mutations in the *TP53* gene also decreased the expressions of pro-apoptotic genes such as *NOXA* or *PUMA* [64] and induced a less intense functional activation of the pro-apoptotic BAX and BAK proteins [65]. The inverse correlation between low BCL2 and high BCL2L1 expression in *TP53*-deficient cells is also observed in pediatric acute lymphoblastic leukemia [66] and more recently confirmed in the Beat AML dataset [67].

Notably, a significant reduction in apoptosis during VEN treatment is observed in *TP53*, *BAX*, or *PMAIP1(NOXA)* KO cells, accompanied by sustained phosphorylation of MAPK1/3 (ERK1/2) and elevated levels of AKT and MAPK1/3 in p53 and BAX-deficient cells. Moreover, the inactivation of *TP53* led to significant alterations in mitochondrial homeostasis, including impaired mitophagy and reduced mitochondrial membrane potential in response to VEN treatment. *TP53* and *BAX* KO cells exhibited enhanced oxidative phosphorylation (OXPHOS) and increased ROS production, indicating a shift in cellular metabolism. These changes suggest that p53 loss compromises mitochondrial stress responses and renders cells resistant to VEN, but they remain susceptible to cell death induced by oxidative stress (i.e., via elesclomol) [52].

It is of interest that unlike the Warburg effect where rapidly proliferating cancer cells in glucose-rich conditions switch to less efficient aerobic glycolysis using the pentose phosphate shunt [68], increased oxygen consumption in *TP53* KO cells suggests an increase in energy production via anaerobic glycolysis in VEN-resistant cells. In resistant MOLM-13 cells with *TP53* and *BAX* inactivation, global metabolomics analysis revealed a decrease in major glycolysis intermediates, amino acids, and urea cycle intermediates, while pyruvate levels remained unaltered, indicating a shift in metabolic pathways. Additionally, these changes were accompanied by increased nucleotide synthesis, validating the altered metabolic states observed in both cell lines and *TP53* mutant patient samples and highlighting the central role of mitochondrial perturbations and apoptotic pathway alterations in VEN resistance [52].

#### 3.3.2. Key Kinases in Venetoclax Resistance: FLT3-ITD and RAS/PTPN11 Mutations

The activation of intracellular signaling pathways by *RAS/PTPN11* or *FLT3* mutant proteins may contribute to VEN resistance. Genomic analysis revealed that patients with *FLT3-ITD* or *PTPN11* mutations either exhibited intrinsic resistance to VEN or developed these mutations during relapse, indicating secondary or acquired resistance [69].

An *FLT3-ITD* mutation confers an adverse prognosis and promotes survival via the activation of the PI3K–protein kinase B (Akt), RAS-MAPK, and STAT5 pathways [70]. Studies have shown that the molecular pathway downstream of FLT3 plays a key role in modulating BCL-xL and MCL1 expression, with STAT5 activation in *FLT3-ITD* mutant cells regulating BCL-xL and Akt influencing MCL1 stabilization [71]. The suppression of STAT5 using siRNA in *FLT3-ITD* mutant cells reduced MCL1 levels, highlighting the interplay between STAT5 and Akt in regulating MCL1 expression [71,72,73]. Similarly, the combination of FLT3 inhibitors with VEN demonstrated strong antileukemic synergy in *FLT3-ITD* AML cell lines and patient samples, with the downregulation of MCL1 and the inhibition of p-ERK being key mechanisms [73]. It is of note that even wild-type FLT3 samples selected based on their poor ex vivo responses to AZA/VEN were found to be enriched for proteins involved in FLT3 pathway signaling, such as RAF/MAP, FLT3, and MAPK1/MAPK3, highlighting a potential mechanism of resistance linked to the activation of FLT3 signaling [74].

*RAS* mutations, particularly in *N/KRAS*, have been identified as a key factor in the development of resistance to VEN in AML [53]. A genomic analysis of primary AML patient samples from the BEAT AML database revealed that *KRAS* or *PTPN11* mutations were associated with higher VEN area under the curve (AUC) values, indicating increased resistance to the drug [38]. In vitro experiments showed that overexpressing KRAS G12D or PTPN11 A72D in AML cell lines recapitulated this resistance. Further analysis revealed that KRAS G12D cells had decreased BCL2 and BAX levels, alongside increased MCL1 and BCL2A1 expression, while PTPN11 A72D cells exhibited elevated MCL1 and BCL-xL levels [38]. Notably, MCL1 inhibitors were effective in reducing cell viability in *KRAS*-mutant cells, while BCL2 inhibition alone or in combination with BCL-xL/BCL-W inhibitors did not achieve similar effects, suggesting that *KRAS*-mutant cells depend on MCL1 for resistance. Similarly, MCL1 inhibition also partially suppressed *PTPN11*-mutant cells, with a limited response to BCL-2/BCL-xL inhibitors [38]. Combining VEN with MCL1 inhibition led to a synergistic effect, overcoming both *KRAS*- and *PTPN11*-mediated resistance [38].

Studies using CRISPR-Cas9-mediated gene editing in human induced pluripotent stem (iPS) cells and primary hematopoietic stem and progenitor cells (HSPCs) show that these mutations emerge later in AML progression. They cause the transformation of granulocyte–monocyte progenitor cells (GMPs), which already harbor other oncogenic mutations. *RAS*-mutant GMP-type LSCs preferentially differentiate into monocytic leukemia cells and display resistance to VEN [55]. Importantly, this resistance is primarily driven by the *RAS*-mutant LSCs themselves, rather than the differentiation state of the LSCs or their progeny. This finding underscores that *RAS* mutations in LSCs play a pivotal role in clinical relapses and the failure of VEN-based therapies, highlighting a new paradigm where specific oncogenic drivers impact the sensitivity of LSCs to targeted treatments, thereby influencing therapeutic outcomes in AML.

### 3.4. Impact of AML Differentiation Status on Venetoclax Resistance

Several preclinical studies have shown that the response of AML to VEN-based therapies varies based on the differentiation status of the blasts, with monocytic differentiation (M5) strongly associated with increased resistance. Ex vivo drug sensitivity testing, guided by flow cytometry, demonstrated a progressive rise in VEN resistance as AML blasts mature from the primitive M0 stage to monocytic M5 [75]. This trend was also supported by the BEAT AML dataset, where AML blasts with high expression of CD14 and CLEA7A, markers typically associated with M4/M5 subtypes, were found to exhibit greater venetoclax resistance [38]. Moreover, a shift in anti-apoptotic protein expression, characterized by a decrease in BCL2 and an increase in MCL1 from M0 to M5, correlated with resistance. The presence of *KRAS* mutations and elevated BCL2A1 expression in M4/M5 AML further suggests their contribution to VEN resistance [38,75]. The analysis of matched patient samples from diagnosis and relapse revealed the coexistence of primitive and monocytic features at diagnosis, with the monocytic clone expanding under venetoclax pressure at relapse while maintaining MCL1 dependency [76]. These findings highlight the complexity of leukemic differentiation and suggest that targeting MCL1 could offer promising strategies to overcome VEN resistance in monocytic AML.

Moreover, recent findings highlight also the connection between erythroid/megakaryocytic differentiation in AML and resistance to VEN [77]. In these rare AML subtypes, cells rely more on the anti-apoptotic protein BCL-xL than BCL2, as demonstrated through ex vivo drug sensitivity testing, genetic perturbation, and transcriptomic profiling. While VEN showed limited efficacy, BCL-xL-selective inhibitors, such as A-1331852 and navitoclax, were highly effective against erythroid/megakaryoblastic leukemia cell lines [77]. High BCL-xL expression in these AML subtypes was confirmed by both single-cell and bulk transcriptomic analyses, contrasting with other subtypes where BCL2 and MCL1 were more prominent. Furthermore, BCL-xL inhibition significantly reduced the tumor burden in a mouse xenograft model, suggesting that targeting BCL-xL could offer a promising therapeutic strategy for erythroid/megakaryoblastic leukemias and highlighting a subgroup of AML with reduced sensitivity to VEN-based treatments [77].

### 3.5. Microenvironment, Metabolism, and Mitochondria in Venetoclax Resistance

Mitochondria are essential organelles involved in cell death, and recent CRISPR screen studies have identified genes related to mitochondrial metabolism and structure that regulate VEN sensitivity in vitro [29,78,79]. An example is CLPB, a gene that maintains mitochondrial cristae structure via its interaction with the OPA1 protein, which is upregulated in AML and further induced upon VEN resistance [79]. Additionally, targeting mitochondrial iron induces a significant loss of mitochondrial respiration, activating BAX/BAK through pathways independent of BH3 mimetics, and synergizes with VEN in both in vitro and in vivo models [78], emphasizing the complex relationship between mitochondrial function and VEN resistance.

Several studies have explored the role of oxidative OXPHOS [80], mitochondrial respiration [81], amino acids [82], and lipids, including fatty acids [83], in their response to VEN. Recent studies have shown that R/R AML following VEN-based treatment, as well as *RAS*-mutated AML, exhibits increased fatty acid metabolism [83]. Additionally, *TP53* mutations, which are linked to VEN resistance, have also been shown to elevate fatty acid levels in AML cell lines [52]. These data suggest that targeting fatty acid metabolism, either through the inhibition of MCL1 or CPT1, can overcome AZA/VEN resistance in LSCs, with MCL1 inhibition affecting both fatty acid and amino acid metabolism. Additionally, increased expression of fatty acid metabolism-related genes correlates with a poor response to AZA/VEN, indicating that fatty acid metabolism may serve as a predictive marker for resistance [83]. Moreover, the integrated stress response (ISR) is strongly investigated, as it might help bridge the gap between cell death, metabolism, and VEN resistance [24,29,78].

Dissecting inflammatory states within the immune microenvironment of AML could reveal mediators of therapeutic resistance and disease progression, as aberrant myeloid cell proliferation leads to the overproduction of pro-inflammatory cytokines that drive leukemic progression [84]. Indeed, a recent study has highlighted the role of enhanced IFNγ signaling, particularly in monocytic differentiation and del7/7q AML samples, as a key factor in VEN resistance in AML [85]. These findings suggest that the immune microenvironment, as well as cellular metabolic pathways, including mitochondrial function and lipid metabolism, significantly influences VEN efficacy, and targeting these pathways could potentially improve treatment outcomes for patients with VEN-resistant leukemia.

## 4. Strategies to Overcome Resistance with New Targeted Combinations

### 4.1. Combining BH3 Mimetics: Conquering the Apoptosis Mechanism

There is growing and increasingly robust preclinical evidence supporting the targeting of the anti-apoptotic protein MCL1, as AML cells depend on it to sustain blast proliferation [10,42,65,86,87]. It has been shown that alterations in the balance of pro- and anti-apoptotic BCL2 proteins, including BAX downregulation, contribute to BH3 mimetic resistance in *TP53*-mutant AML [88]. Co-targeting BCL2 and MCL1 overcomes this resistance, restoring apoptosis in AML and AML stem/progenitor cells both in vitro and in vivo in mouse models [88]. The phase 1, first-in-human MCL1 inhibitor trial of AZD5991 in patients with AML or myelodysplastic syndromes (MDSs) showed a high incidence of troponin elevation and a low overall response rate, with dose-limiting toxicities and four treatment-related deaths [89]. Building on a solid preclinical foundation, additional clinical trials for MCL1 inhibitors are currently underway (NCT03672695, NCT02979366, and NCT02675452), and the scientific community is eagerly anticipating their results (Table 1).

An interesting ongoing phase I clinical trial is combining navitoclax, an early BH3 mimetic targeting not only BCL2 but also MCL1 and BCL-Xl, with AZA/VEN (NCT05222984) to treat AML patients previously treated with VEN-based therapy. Lastly, new dual BCL2/BCLX-xL inhibitors, such as AZD4320 [90], are being developed alongside VHL-recruiting proteolysis-targeting chimeras (PROTACs) [91], which induce the degradation of BCL2 and BCL-xL by recruiting the von Hippel–Lindau E3 ubiquitin ligase. This approach minimizes on-target platelet toxicity, a common issue with navitoclax, as platelets lack VHL expression.

### 4.2. Targeting Epigenetics: Novel Drugs and Approaches

In July 2020, regarding ASTX727 or cedazuridine/decitabine, a fully absorbable oral formulation of DAC was approved for HMA therapy in MDS and CMML [92]. This formulation can now be used in AML, enabling the delivery of a fully oral regimen that includes DAC, VEN, and other targeted oral therapies. Indeed, various clinical trials are ongoing to evaluate either combination with VEN alone (NCT04746235 and NCT04657081) or triplets (NCT05360160, NCT04774393, and NCT05010122). Unlike oral AZA, this oral DAC formulation offers better absorption, and a fully absorbable version of AZA may be available soon [93]. Moreover, it was recently shown that sustained DNA methyltransferase 1 inhibition with frequent weekly lower doses of DAC combined with VEN shows an 88% overall response rate in AML, MDS, and CMML, compared to 64% of the cohort treated with standard dosing HMA/VEN [36]. It is of note that patients with *TP53* mutations achieved an encouraging composite CR rate of 71% and a median OS of 10.7 months [36]. These data indicate that sustained DNA methyltransferase 1 inhibition with frequent lower doses of HMA may offer a strategy to overcome VEN resistance, especially in the difficult-to-treat *TP53*-mutated AML.

Chidamide, a selective histone deacetylase (HDAC) inhibitor, has demonstrated promising effects in combination with AZA/VEN to overcome resistance in AML. Preclinical studies have shown that chidamide downregulates the MCL1 protein, thereby enhancing the efficacy of VEN [94]. In patients with R/R AML, chidamide combined with AZA/VEN improved outcomes for those resistant to VEN, suggesting a potential therapeutic strategy. Additionally, chidamide has shown synergistic effects with AZA, inhibiting leukemia cell proliferation and inducing apoptosis through the downregulation of BCL2 and MCL1 while also promoting myeloid differentiation [95]. These findings highlight the potential of combining HDAC inhibitors with AZA/VEN to overcome resistance and enhance therapeutic efficacy, and various clinical trials are assessing their effectiveness, either as frontline AML treatment (NCT05566054 and NCT06386302) or as salvage in R/R and VEN-resistant disease (NCT05305859 and NCT06220162).

Lysine-specific demethylase 1 (LSD1) is an enzyme that plays a crucial role in regulating gene expression by removing methyl groups from histone proteins, particularly H3K4me2, a marker associated with active transcription. The inhibition of LSD1 using iadademstat leads to the accumulation of H3K4me2 on target genes, promoting blast differentiation and reducing the self-renewal capacity of LSCs, particularly in MLL/KMT2A-rearranged AML [96,97]. Preclinical data show that iadademstat synergizes with standard therapies and enhances the effectiveness of BCL2 inhibition in vitro [96], and clinical trials are investigating its combination with AZA/VEN in AML (NCT06357182 and NCT06514261).

### 4.3. FLT3 and IDH Inhibitors: Double Attack in the Molecular Era

The combination of VEN with FLT3 inhibitors is under rigorous clinical investigation for FLT3-mutated and/or R/R AML. Preclinical and clinical studies have demonstrated significant synergistic effects when VEN is combined with FLT3 inhibitors such as gilteritinib, midostaurin, and quizartinib [70,74,98,99]. In FLT3-mutated R/R AML, VEN plus gilteritinib has shown an impressive complete response (CR) rate of 75% and a median OS of 10 months, even in patients previously treated with FLT3 inhibitors [100], and the “triplet” of AZA/VEN and gilteritinib showed high complete remission rates and deep molecular responses in newly diagnosed FLT3-mutated AML (Table 2) with manageable myelosuppression [101]. However, in the R/R FLT3-mutated AML setting, this “triplet” regimen yields a CR rate of only 27% [101]. Preclinical data show that the combination of gilteritinib with VEN synergistically enhances apoptosis and reduces viability in both AZA/VEN-resistant cell lines and primary patient samples, even in wild-type FLT3 AML, by decreasing MCL1 levels [74]. Thus, future clinical trials might be interrogating the response of the combination in AML even without the *FLT3* mutation. Midostaurin combined with AZA/VEN has proven high response rates; however, significant hematological toxicities are of concern [102,103]. Additionally, in a phase I/II study presented at the ASH meeting in 2023, the triplet regimen of quizartinib, VEN, and DAC has demonstrated a promising composite CR rate of 69% and a median OS of 7.1 months in R/R patients [104]. These results highlight the potential of combining VEN with FLT3 inhibitors to improve outcomes in *FLT3*-mutated AML and beyond, with ongoing or completed trials of gilteritinib (NCT05520567, NCT06317649, and NCT06696183) and quizartinib (NCT03735875, NCT03661307, and NCT04687761) eagerly awaiting updated results.

In line with the concept of double targeting in the molecular era and after validating the efficacy of combining AZA with the IDH1 inhibitor ivosidenib in the phase III AGILE study [105], triplet regimens with IDH inhibitors were further evaluated. The phase Ib/II study of ivosidenib with VEN, with or without AZA in *IDH1*-mutated myeloid malignancies (NCT03471260), showed a promising 90% composite CR rate and a median OS of 42 months for the “triplet” cohort [106]. The combination of the IDH2 inhibitor enasidenib with AZA/VEN has been evaluated in AML patients with *IDH2* mutations, showing promising response rates [107], and further trials assessing the clinical benefit of adding IDH2 inhibitors to AZA/VEN are needed, as durable remissions in *IDH2*-mutated AML are accompanied by variable molecular persistence [56].

**Table 2:** Selected clinical outcomes/preliminary results from VEN-based combination trials in AML.

Drug Regimen		Identifier Clinicaltrias.gov	Eligibility Criteria Other than AML	Number of Patients	Response Rates	Study Phase	Reference
	Exportin 1 (XPO1) inhibitors						
Selinexor + VEN		NCT03955783		19 R/R AML	21% ORR (11% CR, 5% MLFS)	Ib	[108]
Selinexor + VA (SAV)		NCT05736965		20 ndAML	91.7% ORR (80% CRc)	II	[109]
	HDAC inhibitors						
Chidamide + VA (VCA)		NCT05305859		53 R/R AML	81% ORR (CRc 72%) after two cycles	II	[110]
	IDH inhibitors						
Ivosidenib + VEN or VA		NCT03471260	IDH1 mutated myeloid malignancies	30 AML (8 R/R)	63% CRc R/R AML, 93% CRc ndAML	Ib/II	[106]
	FLT3 inhibitors						
Quizartinib + DAC + VEN		NCT03661307	FLT3mut	50 (40 R/R)	69% CR R/R AML	I/II	[104]
Gilteritinib + VEN		NCT03625505	FLT3mut	61 R/R AML	75% mCRc	Ib	[100]
Gilteritinib + VA		NCT04140487	FLT3mut	52 (22 R/R)	96% CRc ndAML, ORR 41% R/R (27% CRc)	I/II	[101]
	MDM2 inhibitors						
Idasanutlin + VEN		NCT02670044		55 R/R or sAML	26% CRc, 12% MLFS	Ib	[111]
	Menin inhibitors						
Revumenib + ASTX727 + VEN		NCT05360160	KMT2Ar, or NPM1c	23 R/R AML	88% ORR (70% CRc)	I/II	[112]
Bleximenib + VA		NCT05453903		45 R/R AML	86% ORR (48% CRc) 82% ORR (36% CRc) with prior VEN exposure	Ib	[113]
	MCL1 inhibitors						
AZD5991 + VEN		NCT03218683	Hematologic malignancies	33 R/R AML	0% ORR		[89]
	CDK inhibitors						
Alvocidib + VEN		NCT03441555		35 R/R AML	ORR 20% (11.4% CRc)	Ib	[114]
QHRD107 + VA		NCT06532058		18 R/R AML	ORR 72.2% (33.3% CRc)	II	[115]
MEK inhibitors							
Trametinib + VA		NCT04487106	RASmut	16 R/R AML	25% ORR (12.5% CRc, 12.5% MLFS)	II	[116]
Cobimetinib + VEN		NCT02670044		22 R/R AML	18% ORR	Ib	[117]
	CD123 targeting molecules						
Tagraxofusp + VA		NCT03113643	AML, MDS, BPDCN	26 ndAML	69% ORR (58% CRc)	I	[118]
	Enhancing drug activity						
ASTX727 + VEN		NCT04746235		62 AML (13 R/R)	64% ORR ndAML, 46% ORR R/R AML	II	[119]
LDDec + VEN		NCT05184842	Myeloid neoplasms	14 AML	63% ORR (57% CRc)	II	[36]
	NEDD8-activating enzyme (NAE) inhibitor						
Pevonedistat + VA		NCT03862157	sAML (MDS/AML)	32 ndAML	78% ORR (66% CRc)	I/II	[120]
Pevonedistat + VA		NCT04172844		50 R/R AML	46.7% ORR AML	I	[121]
	Others						
Magrolimab + VA		NCT04435691			80% ORR ndAML, 34.4% ORR R/R AML (27.5% CRc) 12% ORR R/R AML prior VEN exposure	Ib/II	[122]
Magrolimab + VA vs. VA		NCT05079230		ndAML	39.7% vs. 42.9% CR	III	[123]

VEN: venetoclax, AZA: azacitidine, VA: venetoclax + azacitidine, KMT2Ar: rearranged KMT2A, NUP98r: rearranged NUP98, NPM1c: NPM1-mutated cytoplasmic, sAML: secondary AML, ndAML: newly diagnosed AML, BPDCN: blastic plasmacytoid dendritic cell neoplasm, RASmut: RAS pathway-activating mutation, FLT3mut: mutated *FLT3*, ORR: overall response rate, CR: complete response, CRc: composite complete response, R/R: relapsed/refractory, MLFS: morphological leukemia-free state, LDDec: once-weekly low-dose decitabine.

### 4.4. Restoring p53 Activity to Promote Apoptosis

Targeting the interaction between the tumor suppressor p53 and the E3 ligase MDM2 is a promising therapeutic strategy for cancers with wild-type or functional TP53. MDM2 negatively regulates p53 by promoting its degradation, and inhibiting this interaction can restore p53 function, leading to tumor cell death [124,125]. Several small molecules have been developed to block the p53-MDM2 interaction, with notable inhibitors including idasanutlin, milademetan (DS3032b), and siremadlin. In a phase 1b trial (NCT02670044), the combination of idasanutlin and VEN demonstrated manageable safety and encouraging activity in 55 patients with R/R-AML or secondary AML (sAML), with a CR rate of 26% [111]. Responses were observed early, with a median duration of response of 3.9 months and a median OS of 5.1 months. Patients with mutations in *IDH1/2* and/or *RUNX1* showed the longest OS, while those with *TP53* mutations had a lower response rate, which was expected due to the dependency on intact p53 for idasanutlin activity [111]. Ongoing studies are evaluating alternative MDM2 inhibitors in AML, including siremadlin in combination with AZA/VEN (NCT05155709) [126] and the newer MDM2 inhibitor KRT-232 in combination with VEN and DAC (NCT03041688).

Recent preclinical studies have highlighted the potential of combining selective inhibitors of nuclear export (SINE) compounds with VEN in AML treatment [127,128]. These drugs target exportin 1 (XPO1), inhibiting the export of tumor suppressor proteins like p53, thereby restoring their function and triggering apoptosis in leukemic cells. Ongoing clinical trials are investigating the efficacy of combining these SINE inhibitors with AZA/VEN in AML (NCT03955783, NCT05736965, NCT06449482, and NCT06399640). It is of interest that in a preclinical setting, the triple inhibition of MDM2, XPO1, and BCL2 effectively overcomes resistance by reactivating p53, disrupting c-MYC-driven resistance, and preventing stress-adapted VEN resistance in AML [129].

### 4.5. Alternative Pathways to Trigger Apoptosis

Various strategies have been explored to indirectly modulate MCL1 to overcome apoptosis resistance, such as suppressing its transcription through cyclin-dependent kinase (CDK) inhibitors [130], enhancing NOXA expression to facilitate its neutralization via NEDD8-activating enzyme (NAE) inhibitors [131], and leveraging MEK or RAS inhibitors to target the RAS-RAF-MEK-ERK (MAPK) pathway [132], thereby promoting MCL1 degradation. The combination of VEN with alvocidib, a potent CDK9 inhibitor, in R/R AML was safe and tolerable but showed only modest efficacy [114], while other CDK inhibitors are under investigation (NCT04017546, NCT06532058, and NCT06191263). Moreover, the combination of AZA/VEN with the MEK inhibitor trametinib, as well as the combination of VEN with the MEK1 inhibitor cobimetinib, showed modest activity in R/R AML, with a response rate of 25% and 18%, respectively, along with substantial toxicity, limiting their clinical use [116,117]. Pevonedistat, an NAE inhibitor, has shown more promising results, as in phase I and I/II trials, in combination with AZA/VEN, it demonstrated composite CR rates of 66% in newly diagnosed sAML and an overall response rate of 46.7% in R/R AML [120,121]. The phase II study evaluating the combination’s efficacy in frontline setting is active (NCT04266795). Lastly, in a preclinical setting, the RAS inhibitor RMC-7977 repressed the phosphorylation of downstream effectors of the MAPK pathway (MEK, ERK, and RSK) and was potent against *FLT3*-ITD- and *RAS*-mutated AML cell lines [133]. Moreover, unlike gilteritinib/VEN combinations that are selected for the survival of cells harboring *NRAS* mutations, RMC-7977 inhibited the outgrowth of all cell populations, highlighting a promising partner for VEN [133].

Targeting the apoptosis of *TP53*-mutated disease has been a big challenge. Eprenetapopt (APR-246) is a low-molecular-weight compound that restores the wild-type function of mutant p53. In addition to targeting mutant p53, eprenetapopt inhibits thioredoxin reductase 1 (TrxR1), converting it to an active oxidase, and depletes cellular glutathione, resulting in increased ROS, contributing to its antileukemic effects. The combination of eprenetapopt with AZA/VEN demonstrated an acceptable safety profile and encouraging activity, with 64% of patients achieving an overall response and 38% of patients with *TP53*-mutated AML achieving CR in a phase I expansion study [134]. However, recent phase III study results released by Aprea Therapeutics show that the combination of eprenetapopt with AZA did not significantly improve CR rates compared to AZA alone, weakening the initial excitement about this approach for *TP53*-mutated disease.

### 4.6. Other Novel Approaches: The Microenvironment in the Spotlight

Targeting the leukemic microenvironment is an emerging strategy in AML treatment. LSCs evade macrophage-mediated phagocytosis by overexpressing CD47, which binds to the SIRPα receptor on macrophages, delivering a “do not eat me” signal. Magrolimab, an anti-CD47 antibody, was the first therapeutic to enter clinical trials targeting this interaction and showed promising results in reducing the LSC fraction without augmenting the toxicity of AZA. Even though the combination of AZA/VEN and magrolimab initially demonstrated promising results in phase I/II trials, along with manageable safety [122], the ENHANCE III study was discontinued as in patients with newly diagnosed AML, the triplet did not improve OS or CR rates [123]. Additionally, the bone marrow microenvironment provides protection and survival signals to AML cells, complicating the efficacy of cytotoxic regimens like VEN. Recent studies have highlighted CD44’s role in mediating VEN resistance through the CXCR4-CXCL12 pathway, and blocking CD44 or CXCR4 could potentially enhance treatment effectiveness, paving the way for new research [135].

Lastly, innovative immunotherapies include anti-CD123 antibody–drug conjugates that are also under investigation for their potential to target specific markers on AML cells, with early clinical results showing promising safety and efficacy profiles (NCT04086264, NCT06456463, NCT05442216, NCT06634394, and NCT06456463).

## 5. Conclusions

In conclusion, the advent of VEN has significantly transformed the treatment landscape for AML, particularly for patients unfit for intensive chemotherapy, with HMA/VEN now serving as the standard of care. The integration of molecular profiling has enhanced our understanding of both the mechanisms of responsiveness and resistance by identifying key mutations that can serve as predictive markers for patient outcomes. *NPM1* mutations are identified as strongly predictive of a favorable response and sustained remission, not only with chemotherapy but also for VEN-based therapies, followed by *IDH2* mutations that emerge as a significant secondary predictor. On the other hand, *TP53* mutations, along with *FLT3-ITD* and *RAS* mutations, are associated with VEN resistance [53]. In the molecular era, the double attack of combining VEN-based treatment with targeted treatment with FLT3 inhibitors and IDH1/2 inhibitors is investigated, with promising results [100,101,104,106]. Preclinical data showing the synergistic apoptotic effect of the gilteritinib/VEN combination even in wild-type FLT3 primary patient samples, as well as their promising clinical outcomes [74] (Table 2), support its exploration in VEN-resistant diseases, regardless of mutational status. To target the RAS-RAF-MEK-ERK (MAPK) pathway, CDK9 and MEK inhibitors are evaluated, with modest activity in preliminary data [114,115,116,117]. Novel RAS inhibitors, such as RMC-7977, repressing the phosphorylation of downstream effectors of the MAPK pathway are potent against *FLT3*-ITD- and *RAS*-mutated AML cell lines [133] and are being highlighted as promising partners for VEN.

Additional factors contributing to VEN resistance include monocytic and erythroid/megakaryocytic differentiation states, the altered expression of other components of the apoptosis mechanism, as well as metabolic reprogramming. Targeting other BCL2 family members, such as MCL1 and BCL-xL, is currently in the spotlight of clinical trials, supported by robust preclinical data [37,41,42]. New dual BCL2/BCLX-xL inhibitors, such as AZD4320 [90], are being developed alongside VHL-recruiting PROTACs [91] that aim to minimize on-target platelet toxicity. Restoring p53 activity to promote apoptosis with drugs targeting XPO1 or MDM2 inhibitors is being investigated, though clinical responses in the R/R setting have been modest to date [108,109,111].

Recently, genome-wide CRISPR/Cas9 screening led to the identification of p53 and BAX as key factors of resistance [52], and it is of interest that 17% of AML patients relapsing after VEN-based therapy have acquired inactivating missense or frameshift/nonsense mutations of *BAX* [51]. The loss or inactivation of *TP53* reduces pro-apoptotic gene expression (e.g., PUMA and NOXA) and alters mitochondrial function, leading to increased BCL-xL expression and resistance to VEN treatment [52,64,65,66,67]. These changes are associated with impaired mitophagy, enhanced OXPHOS, and metabolic reprogramming, including increased ROS and nucleotide synthesis, and preclinical data suggest that TP53-deficient cells evade apoptosis but remain vulnerable to oxidative stress-induced death [52,68]. A rational and promising direction for future research involves investigating metabolic dependencies and the role of the bone marrow microenvironment, with the aim of identifying and targeting actionable metabolic vulnerabilities through rational drug combinations that can restore VEN sensitivity in patients with a higher risk of AML.

Ongoing clinical trials are investigating various strategies to address the challenges of resistance and response durability. Approaches such as “doublet” and “triplet” therapies aim to deepen initial responses and prevent mutation emergence, though their effectiveness may be limited to specific genomic subsets and require careful dose adjustments of VEN. Furthermore, adaptive sequential therapy shows potential, but its success hinges on the availability of advanced molecular techniques to detect emerging clones early. Looking ahead, comprehensive molecular profiling, including single-cell DNA and RNA sequencing, holds the potential to deepen our understanding of clonal evolution and dynamic pathway rewiring in the context of resistance, thereby enabling the design of patient-specific post-VEN therapeutic strategies that can overcome resistance and improve clinical outcomes for HMA/VEN-refractory AML.

## Figures and Tables

**Table 1:** Ongoing clinical trials of Venetoclax-based targeted combination regimens in AML.

Drug Regimen	Identifier Clinicaltrias.gov	Eligibility Criteria Other than AML	Treatment Category	Study Phase	Year of Initiation
Exportin 1 (XPO1) inhibitors					
Selinexor + VEN	NCT03955783	AML or DLBCL	Salvage	Ib	2019
Selinexor + VA (SAV)	NCT05736965		Frontline	II	2023
SAV	NCT06449482		Frontline	I/II	2023
Eltanexor + VEN	NCT06399640	AML or MDS	Salvage	I	2024
HDAC inhibitors					
Chidamide + VA (VCA)	NCT05305859		Salvage	II	2022
VCA	NCT05566054		Frontline	II	2022
VCA	NCT06220162	Failed response to VA	Salvage	II	2024
VCA	NCT06386302		Frontline	II	2024
IDH inhibitors					
Ivosidenib + VEN or VA	NCT03471260	IDH1 mutated myeloid malignancies	Salvage	Ib/II	2018
ASTX727 + VEN + IDH inhibitor	NCT04774393		Salvage	Ib/II	2021
Ivosidenib + VEN	NCT06611839	IDH1 mutation	Both	I/II	2024
FLT3 inhibitors					
Quizartinib + DAC + VEN	NCT03661307		Both	I/II	2018
Gilteritinib + VA	NCT04140487	FLT3mut CMML, MDS/MPN, AML	Salvage	I/II	2019
Quizartinib +VA or (LDAC + VEN) (VEN-A-QUI trial)	NCT04687761		Frontline	I/II	2020
ASTX727 + gilteritinib + VEN	NCT05010122	FLT3mut	Both	I/II	2021
Gilteritinib + VA	NCT05520567	FLT3mut	Frontline	I/II	2023
Gilteritinib + VA vs. VA	NCT06317649	FLT3mut	Frontline	II	2024
Gilteritinib + VA	NCT06696183	FLT3mut	Frontline	II	2025
MDM2 inhibitors					
KRT-232 + DAC + VEN	NCT03041688		Both	Ib	2018
Siremadlin + VA	NCT05155709		Both	Ib/II	2022
Menin inhibitors					
Revumenib + ASTX727 + VEN	NCT05360160	KMT2Ar, NUP98r, or NPM1c	Both	I/II	2022
Revumenib + VA	NCT06652438	KMT2Ar, or NPM1c	Frontline	III	2024
Bleximenib + VA	NCT05453903		Frontline	Ib	2022
MCL1 inhibitors					
AMG176 +/− VEN	NCT02675452	AML or MM	Salvage	I	2016
S64315 + VEN	NCT03672695		Salvage	I	2018
CDK inhibitors					
Alvocidib + VEN	NCT03441555		Salvage	Ib	2018
CYC065 + VEN	NCT04017546		Salvage	I	2019
QHRD107 + VA	NCT06532058		Salvage	II	2023
RVU120 + VEN (RIVER-81 trial)	NCT06191263		Salvage	II	2024
PD1 inhibitors					
Pembrolizumab + DAC +/− VEN	NCT03969446	AML or MDS	Both	Ib	2020
AK117 + VA	NCT06387420		Both	I	2024
Tislelizumab + VA	NCT06536959	AML or MDS	Salvage	II	2024
CD123 targeting molecules					
IMGN632 + VA	NCT04086264		Salvage	Ib/II	2019
Tagraxofusp + VA	NCT06456463	CD123^+^	Frontline	II	2022
Tagraxofusp + VA	NCT05442216	sAML post HMA	Salvage	II	2024
APVO436 + VEN (RAINIER trial)	NCT06634394	CD123^+^ AML	Frontline	Ib/II	2024
Tagraxofusp + VA	NCT06456463	CD123^+^ AML	Frontline	II	2025
Enhancing drug activity					
ASTX727 + VEN	NCT04746235		Both	II	2021
ASTX727 + VEN	NCT04657081		Frontline	I/II	2021
Cobicistat + VA	NCT06014489		Frontline	II	2024
NEDD8-activating enzyme (NAE) inhibitor					
Pevonedistat + VA vs. VA	NCT04266795		Frontline	II	2020
Others					
Lintuzumab + VEN	NCT03867682		Salvage	I/II	2020
Cusatuzumab + VA	NCT04150887		Frontline	Ib	2019
Cusatuzumab + VA	NCT06384261		Frontline	II	2024
evorpacept + VA (ASPEN-05)	NCT04755244		Both	Ι/ΙΙ	2021
Navitoclax + VA	NCT05222984	VEN treated AML	Salvage	I	2022
Iadademstat + VA	NCT06357182		Frontline	Ib	2024
Iadademstat + VA	NCT06514261		Frontline	I	2025

VEN: venetoclax, AZA: azacitidine, VA: venetoclax + azacitidine, KMT2Ar: rearranged KMT2A, NUP98r: rearranged NUP98, NPM1c: NPM1-mutated cytoplasmic, MM: multiple myeloma, sAML: secondary AML, ndAML: newly diagnosed AML, BPDCN: blastic plasmacytoid dendritic cell neoplasm, RASmut: RAS pathway-activating mutation, FLT3mut: mutated *FLT3*.

## Data Availability

No new data were created or analyzed in this study. Data sharing is not applicable to this article.
